# Upregulation of Peridinin-Chlorophyll A-Binding Protein in a Toxic Strain of *Prorocentrum hoffmannianum* under Normal and Phosphate-Depleted Conditions

**DOI:** 10.3390/ijms24021735

**Published:** 2023-01-15

**Authors:** Thomas Chun-Hung Lee, Kaze King-Yip Lai, Steven Jing-Liang Xu, Fred Wang-Fat Lee

**Affiliations:** 1 School of Science and Technology, Hong Kong Metropolitan University, Hong Kong, China; 2 State Key Laboratory of Marine Pollution, City University of Hong Kong, Kowloon, Hong Kong, China

**Keywords:** diarrhetic shellfish poisoning (DSP), *Prorocentrum*, harmful algae, proteomics

## Abstract

Some strains of the dinoflagellate species *Prorocentrum hoffmannianum* show contrasting ability to produce diarrhetic shellfish poisoning (DSP) toxins. We previously compared the okadaic acid (OA) production level between a highly toxic strain (CCMP2804) and a non-toxic strain (CCMP683) of *P. hoffmannianum* and revealed that the cellular concentration of OA in CCMP2804 would increase significantly under the depletion of phosphate. To understand the molecular mechanisms, here, we compared and analyzed the proteome changes of both strains growing under normal condition and at phosphate depletion using two-dimensional gel electrophoresis (2-DE). There were 41 and 33 differential protein spots observed under normal condition and phosphate depletion, respectively, of which most were upregulated in CCMP2804 and 22 were common to both conditions. Due to the lack of matched peptide mass fingerprints in the database, de novo peptide sequencing was applied to identify the differentially expressed proteins. Of those upregulated spots in CCMP2804, nearly 60% were identified as peridinin-chlorophyll a-binding protein (PCP), an important light-harvesting protein for photosynthesis in dinoflagellates. We postulated that the high expression of PCP encourages the production of DSP toxins by enhancing the yields of raw materials such as acetate, glycolate and glycine. Other possible mechanisms of toxicity related to PCP might be through triggering the transcription of non-ribosomal peptide synthetase/polyketide synthase genes and the transportation of dinophysistoxin-4 from chloroplast to vacuoles.

## 1. Introduction

Humans who consume shellfish contaminated with phycotoxins are subject to different types of shellfish poisoning. Dinoflagellates are one of the primary sources to produce these toxins, which accumulate in shellfish when they filter-feed on toxic algae and it cannot be destroyed through cooking to normal internal temperatures [1,2]. Despite not being the most severe type of shellfish poisoning, diarrhetic shellfish poisoning (DSP) has received considerable attention to the sectors of public health, food safety and the shellfish industry after several outbreaks occurred worldwide in the last decade [3,4,5,6]. Almost all cases of DSP reported were associated with the consumption of contaminated mussels harvested from marine areas where harmful species of the dinoflagellates *Dinophysis* or *Prorocentrum* were found [3,4,5,7,8]. A systematic scheme for monitoring DSP toxins in harvested shellfish, together with harmful microalgae in shellfish harvesting zones, would undoubtedly help farmers to identify possible contamination and take precautionary measures. To facilitate the monitoring process, several screening tools and detection methods have been developed and their performance was tested [9,10,11,12,13,14,15,16,17]. However, in the long run, it is even more important to extend our knowledge about the biosynthesis of DSP toxins in those toxic dinoflagellates so that we may mitigate their production in the first place. In the past, feeding studies through the incorporation of isotope-labelled precursors to dinoflagellates provided some insights on the synthesis of okadaic acid (OA) and dinophysistoxins (DTXs) [18,19,20,21,22]. These feeding experiments, however, are not effective and powerful enough to unravel complex biosynthetic pathways. With omics technologies becoming more popular, we can now discover genes that are potentially related to dinoflagellate toxicity and elucidate the mechanisms of toxin biosynthesis. Since cellular levels of toxins in dinoflagellates have been shown to be greatly affected by nutritional and environmental factors [23], we believe that by examining the functional gene products rather than the genetic code alone through proteomics, a more comprehensive picture of the DSP toxin biosynthetic pathways could be obtained.

To date, the majority of proteomic studies on dinoflagellates focused on the biosynthesis of paralytic shellfish poisoning (PSP) toxins in *Alexandrium* spp. but scarcely DSP toxins [24,25,26,27,28,29,30,31]. These proteomic studies compared the two-dimensional gel electrophoresis (2-DE) profiles between two strains with dramatic difference in cellular toxicity or between two cultures with dramatic variation of toxicity triggered by different environmental conditions or growth phases [24,25,29,30,31]. After a comparison of the 2-DE profiles, differential protein spots were subjected to de novo peptide sequencing using MS/MS and N-terminal sequencing. In comparison with other toxic dinoflagellates, the identification of proteins involved in PSP toxin biosynthesis is highly feasible because cyanobacteria including *Anabaena*, *Aphanizomenon*, *Cylindrospermopsis* and *Lyngbya* are also PSP toxin producer. The gene clusters of toxin biosynthesis in these cyanobacteria were well characterized [32]. Recent study also demonstrated that a species of *Alexandrium* shared some cyanobacterial proteins for PSP toxin biosynthesis.

We compared the growth and toxicity responses between two closely-related strains of *P. hoffmannianum* (CCMP2804 and CCMP683) in our previous study [33]. OA but not DTX-1 and DTX-2 was detected in CCMP2804 (6.31 pg cell^−1^ on day 28 and 12.37 pg cell^−1^ on day 50). The cellular concentration of OA in CCMP2804 increased significantly from 12.37 pg cell^−1^ to 67.03 pg cell^−1^ on day 50 when cells were cultivated without the supply of phosphate. However, no OA and other DSP toxin derivatives could be detected on CCMP683 at any culturing conditions examined. In this study, comparative proteomics was performed based on identification of differentially expressed proteins in the 2-DE profiles between the toxic strain (CCMP2804) and the non-toxic strain (CCMP683) of *P. hoffmannianum* cultured with normal nutrient supply until log phase for 28 days (log) and under phosphate depletion for 50 days (0P).

## 2. Results and Discussion

### 2.1. Differential Protein Expression between Toxic and Non-Toxic Strains

#### 2.1.1. Normal Culture Condition

**Figure 1:**
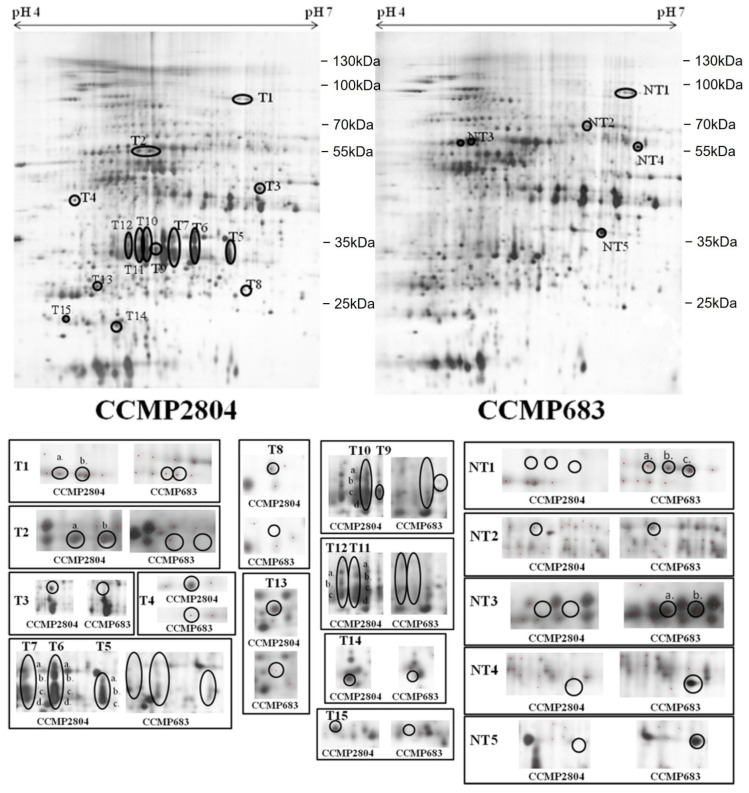
Representative 2-DE profiles of 100 μg protein extracts from the two strains of *P. hoffmannianum* with normal nutrient supply separated over a pH range of 4–7 and then by 10% SDS-PAGE. Protein visualization was performed with silver stain. The circled spots with initials “T” and “NT” were upregulated in the toxic strain CCMP2804 and non-toxic strain CCMP683 respectively when compared with each other. A side-by-side comparison of each pair of enlarged differential protein spots was included.

In order to search for differentially expressed proteins between the toxic and non-toxic strains of *P. hoffmannianum*, vegetative cells of CCMP2804 and CCMP683 grown in normal L1 medium without sodium silicate (L1-Si medium) were harvested for 2-DE analysis on day 28 when maximum growth rates of both strains were observed [33]. Equal amounts of protein extracts were loaded onto each 2-DE gel. The experiment was performed in triplicate. The 2-DE gels after silver staining were scanned into computer and analyzed by using Melanie 7. Figure 1 depicts the corresponding position and the intensity of each spot in the representative 2-DE profiles of CCMP2804 and CCMP683 respectively. The isoelectric point (pI), molecular weight (MW) and the fold change of each differential spot was summarized in Table S1. Approximate 560 spots matched between the two strains, indicating that the identities of the two strains are very close. Previously, species specific 2-DE profiles were observed in Chan’s study and could act as proteome reference maps for species identification even in *A. tamarense* complex [28,34]. Of these approximately 560 protein spots, 41 spots appeared differentially expressed by at least two folds between the two strains during log phase. Among these spots, 33 protein spots were up regulated in the protein profile of CCMP2804 whereas 8 spots were up regulated in that of CCMP683. Fifteen spots were uniquely found among those 33 upregulated spots in the 2-DE profile of CCMP2804, while four out of eight upregulated spots were only expressed in that of CCMP683.

#### 2.1.2. Phosphate Depletion

**Figure 2:**
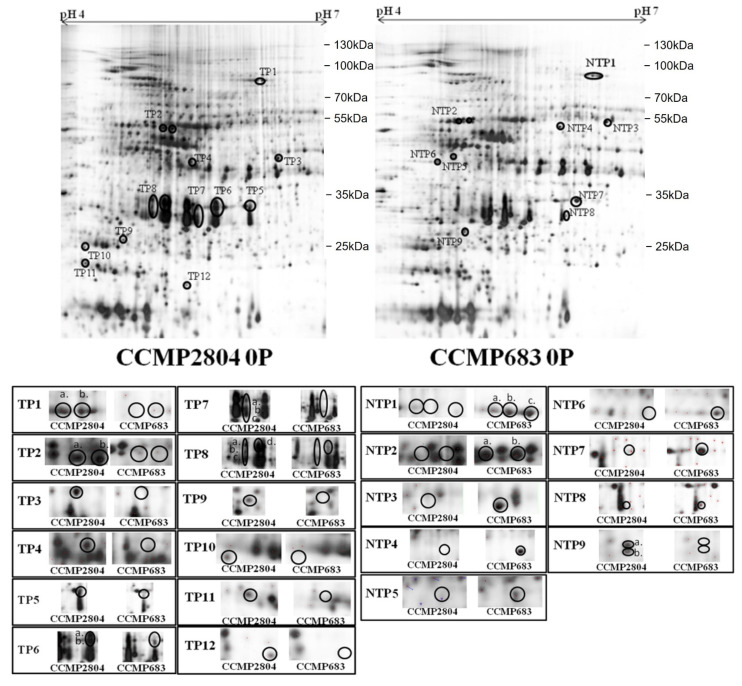
Representative 2-DE profiles of 100 μg protein extracted from CCMP2804 and CCMP683 under phosphate depletion (0P) separated over a pH range of 4–7 and then by 10% SDS-PAGE. Protein visualization was performed with silver stain. The circled spots with initials “TP” and “NTP” were upregulated in the toxic strain and non-toxic strain respectively when compared with each other. A side-by-side comparison of each pair of enlarged differential protein spots was included.

In our previous study, the maximal increment in cellular toxicity was found in culture of CCMP2804 under 0P whereas CCMP683 still remained non-toxic [33]. Therefore, proteomic analysis was performed to explore the proteome changes of the algal cells growing under the 0P condition. The same amount of protein extracts and approach mentioned in the previous section was used. The corresponding position and intensity of each differential spot were labelled in the representative 2-DE profiles of CCMP2804 and CCMP683, respectively (Figure 2). The pI, MW and the fold change of each differential spot are also summarized (Table S2). Similar to the samples during log phase, over 500 spots were successfully matched between the two strains. In total, 33 protein spots were differentially expressed by two folds between the cultures of both strains under 0P. Twenty spots were upregulated in 2-DE profile of CCMP2804 under 0P whereas 13 spots were upregulated in that of CCMP683. Among these spots, 10 out of 20 spots were uniquely expressed in the 2-DE profiles of CCMP2804 while 10 out of 13 spots were only expressed in the 2-DE profiles of CCMP683.

### 2.2. Shared Proteins between Both Strains during Log and under 0P

**Figure 3:**
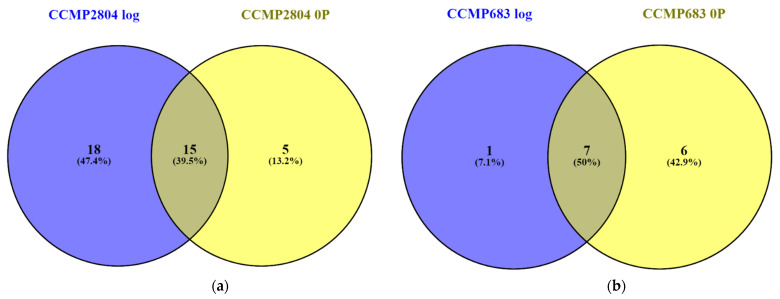
Number of protein spots showing at least two-fold differences in intensity between CCMP2804 and CCMP683 with normal nutrient supply in log phase (log) versus under 0P on day 50: (**a**) upregulated spots in CCMP2804; (**b**) upregulated spots in CCMP683. The area of overlapping shows differential spots commonly found during log and under 0P.

**Figure 4:**
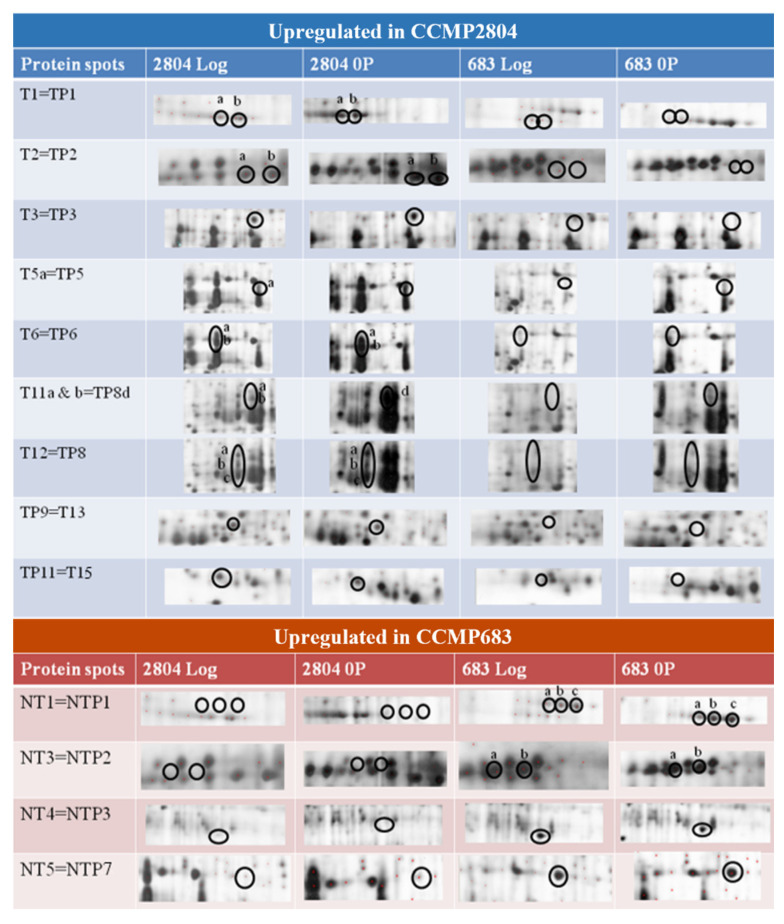
Upregulated protein spots that were commonly found during log and under 0P in the 2-DE profiles of CCMP2804 or CCMP683 when compared to each other.

After several comparisons of the 2-DE profiles, the spots uniquely observed in the 2-DE of CCMP2804 or CCMP683 were summarized (Tables S1 and S2). Regardless of the conditions, totally 15 protein spots were consistently upregulated in the 2-DE profile of CCMP2804 while seven protein spots also were consistently upregulated in that of CCMP683 (Figure 3 and Figure 4). The differentially expressed spots between log phase cultures of both strains might belong to proteins related to toxin biosynthesis and cell growth because the results in our previous study showed that non-toxic CCMP683 had a longer lag phase and relatively lower maximum cell density even though they have the same growth rate [33]. The differentially expressed spots between cultures of both strains under 0P indicated that these proteins may be involved in toxin biosynthesis because both strains could not grow rapidly in this condition. In other words, 15 shared proteins in CCMP2804 probably involved and encouraged the DSP toxin production but seven shared proteins in CCMP683 may play a role in suppression of the DSP toxin production.

### 2.3. Identification of Differentially Expressed Proteins

Peptide mass fingerprints (PMFs) from all the differentially expressed spots were searched against the National Center for Biotechnology Information (NCBI) database using MASCOT. Nevertheless, most of the spots could not be identified through this method. This could be attributed to the lack of sufficient genome and proteome database information for dinoflagellates. Searching for protein identities from those protein or DNA sequences submitted to public databases was the only approach without genome and proteome databases of dinoflagellates. Even though the proteins had the same function, the identities of proteins still could not be identified due to inadequate homology of the protein sequence between information in database and target protein.

Since protein identification based solely on PMFs was insufficient, the amino acid sequence tags from selected peptides of each PMF were obtained by using MS/MS. Moreover, the MS/MS mode was also performed with sulphonation in order to aid the de novo sequencing of the selected peptide by minimizing the interference from ions of other series. Once the peptide was successfully sulphonated, the sequence of the target peptide could be easily deduced from the mass difference between mass peaks in the MS/MS spectrum as sulphonation promotes the fragmentation of y-type ions in peptide. Such a method has been successfully applied in the sequencing of peptides in *Alexandrium* species [35]. Peptide sequences on protein spots T4, T8, T9, T15, NT2, TP4, TP11, TP12, NTP5, NTP6 and NTP8 failed to be determined by the MS/MS analysis. One of the main reasons was that these spots lacked adequate quantity of proteins for the MS/MS peptide sequencing. Some of the differential spots were successfully determined with at least seven successive amino acid in de novo peptide sequencing but they failed to be identified when searching the database of NCBI and UniProt using MASCOT and BLAST.

Ultimately two protein spots were successfully identified. The first one is T2b and it only appeared in the 2-DE profiles of CCMP2804. The MS/MS spectrum of one of the peptides *m*/*z* 1229.6 significantly matched the putative uncharacterized protein of *Noctiluca scintillans* (UniProt entry: A7WQ96|A7WQ96_NOCSC) with sequence LAMLEALSNLR. Another protein spot, T6a, was identified with sequence tags (mass peaks *m*/*z* 1258.6 and *m*/*z* 2393.2) generated from the MS/MS spectrum (Figure S1). T6a was upregulated by two folds or above in the 2-DE profile of CCMP2804 during log and under 0P (i.e., TP6a) (Tables S1 and S2). These peaks were selected for further fragmentation by using MS/MS mode and amino acid residues were then deduced from the mass difference between the fragmented ions. After MASCOT bioinformatic search, a high score indicated that the deduced sequences significantly matched the peridinin chlorophyll-a binding protein (PCP) of *Symbiodinium* sp. (accession no.: AAN39418) with the sequences ADWDAVNAALGR and AIGSISGPNGVTSRADWDAVNAAIGR of mass peaks *m*/*z* 1258.6 and *m*/*z* 2393.2 respectively. Interestingly, many spots were also identified as PCP as their PMF spectra were highly similar and all spectra contained the mass peaks of *m*/*z* 1258.6 and *m*/*z* 2393.2 (Table 1). From the 2-DE of CCMP2804 during log and under 0P, these groups of proteins were exhibited as spot trains with pI and MW ranging approximately from 6.1 to 5.1 and from 34 to 29 kDa, respectively. Thus, these proteins are probably the isoforms of PCP. Their variation in pI and MW could be attributed to post-translational modifications, which are quite common in dinoflagellates [36,37,38].

### 2.4. Possible Implications of Identified Proteins to DSP Toxin Production by P. hoffmannianum

The identified proteins and their expression were summarized in Table 1. The mass peak *m*/*z* 1229.6 of spots T2b was identified as putative uncharacterized protein and thus, function of the protein could not be deduced. The protein identity of spots T5–7, T10–12 and TP5–8 was confirmed as PCP by using MASCOT bioinformatics search after de novo peptide sequencing. PCP was a water-soluble protein. This protein is generally believed to be located in thylakoid lumen of photosynthetic dinoflagellates [39,40] and works as one of the major light-harvesting complexes. The primary pigment, carotenoid peridinin absorbs blue-green light from 470 nm to 550 nm [41]. The PCP of dinoflagellates is very diverse in structure, MW, pI, amino acid sequence, ratio between peridinin and chlorophyll a and spectroscopic properties among different species and even strains. The MW of PCP is dependent on the form of PCP, with the homodimer form being 14–16 kDa while the monomer form being 30–35 kDa [42,43]. Within the monomer form, it can be further divided into a main form PCP and a high salt PCP with MW of 32 kDa and 34 kDa, respectively [44]. The MW of the PCP identified in this study is close to those of the monomer forms including both the major form PCP and the high salt PCP. Until now, our understanding of the functions of different forms of PCP is still very limited. A previous study reported that the protein sequences between the major form PCP and high salt PCP only shared approximately 30% similarity, resulting in peridinin loss and chlorophyll phytol chains rearrangement in the high salt PCP [45]. Moreover, the pI of the PCP identified is slightly acidic with a range from 5.1 to 6.1, which is similar to the PCP of *Gonylaulax polyedra* (pI~6) but different from the slightly basic or less acidic PCP of *Amphidinium carterae* (pI~7.5), *S. microadriaticum* (pH 7.2–7.7) and *A. catenella* (pH 6.2–6.9) [42,46,47].

A significantly higher expression level of PCP (spots T5–12) found in CCMP2804 during the log phase could enhance the efficiency of photon capture under light [48]. Consequently, the increase in photosynthetic rate may promote the fixation of carbon dioxide, metabolism, generation of energy and synthesis of vital materials for cell growth. Some PCP isomers including T6–7, T10–12 and TP6–8, were significantly upregulated or only expressed in CCMP2804 (Tables S1 and S2). This indicates that DSP toxin biosynthesis might be related to light harvesting. Pan et al. reported that the DSP toxin production of *P. lima* was paused during 21-day incubation in continuous darkness and proved that light is an essential component in DSP toxin production [49]. In fact, we once postulated a pathway of DSP toxin production based on the literature, in which light might encourage toxin production through triggering the transcription of hybrid non-ribosomal peptide synthetase/polyketide synthase (NRPS/PKS) gene and transportation of DTXs from chloroplasts to vacuoles [50]. DSP toxins were suggested to be synthesized by hybrid NRPS/PKS located in chloroplasts due to their polyketide structures [51]. The expression of NRPS/PKS gene is probably controlled by light because the expression of partial transcripts, *mcyB* and *mcyD*, in cyanobacterial NRPS/PKS gene was shown to be regulated by the presence of light [52]. In this study, specific isomers of PCP in CCMP2804 were probably responsible for activating the transcription of NRPS/PKS gene by capturing more light. Almost all excitation energy from peridinin was shown to be transferred to chlorophyll a and then unknown light-energy conversion machineries that turn energy into signals for transcription [41,53]. Besides, DTX-4 was suggested to be transported from chloroplasts and vacuoles by a light mediated mechanism, as revealed by immunolabeling studies showing that DSP toxins are observed in both chloroplasts and vacuoles especially at the cellular periphery [54,55]. This light mediated mechanism might be triggered by the extra light energy captured by upregulated PCP in CCMP2804.

These specific PCP isomers might also influence the production of raw materials for DSP toxin biosynthesis. Several precursor incorporation studies revealed that OA and the side chain of DTXs are synthesized from glycolate following by the consecutive addition of acetate units [18,56,57]. Moreover, the intact glycine molecule located in the side chain of DTX-5b indicated that hybrid NRPS/PKS utilized glycine for mid chain incorporation [19]. Thus, glycolate, acetate and glycine are the raw materials for DSP toxin biosynthesis. Although the mechanism of acetate synthesis in algae was still unknown, in the bacterial species *Clostridium thermoaceticum*, acetyl coenzyme A is synthesized from carbon dioxide and coenzyme A by an enzyme called carbon monoxide dehydrogenase [58]. As the upregulation of PCP increases the photosynthetic rate, it is believed that the production of acetate also increases with the enhanced rate of carbon dioxide fixation. The starter unit of OA biosynthesis glycolate and the mid chain incorporating unit glycine are probably generated from a light-oriented reaction called photorespiration, which was found in various algae in more than 30 years ago [59,60]. In brief, oxygen is fixed with ribulose 1,5 bisphosphate by ribulose-1,5-bisphosphate carboxylase/oxygenase to form phosphoglycolate, which is then dephosphorylated by phosphoglycolate phosphatase to become glycolate. Glycolate could be oxidized to glyoxylate by glycolate oxidase and gets transaminated to become glycine by serine glyoxylate aminotransferase. The enhanced efficiency of light capturing by upregulated PCP may increase the rate of photorespiration, and eventually the production of glycolate and glycine for DSP toxin biosynthesis.

**Figure 5:**
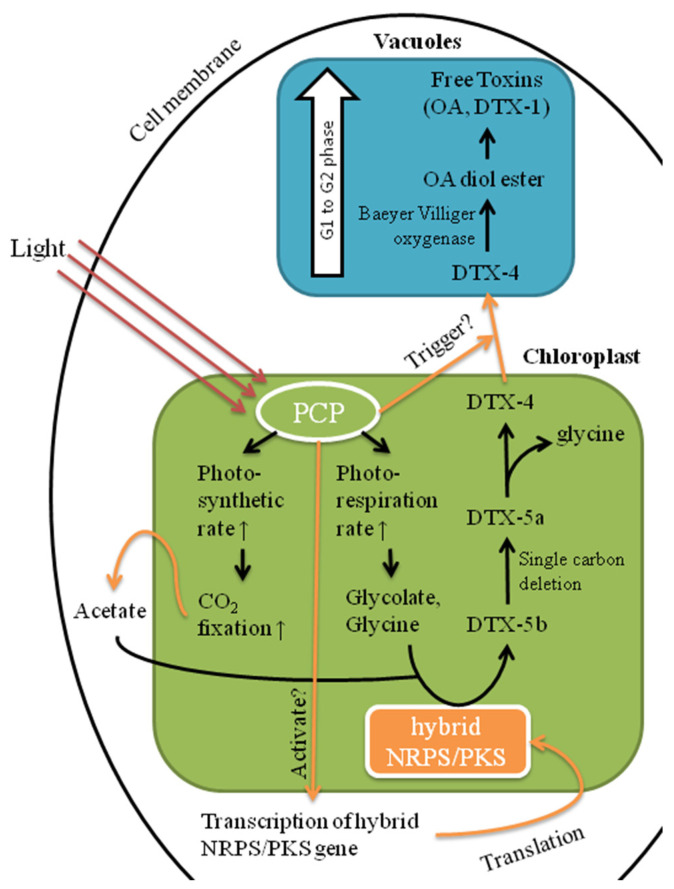
The possible pathway of DSP toxin biosynthesis and the role of peridinin chlorophyll a binding protein (PCP) and hybrid NRPS/PKS. Orange arrows indicate possible pathways suggested by this study while black arrows indicate demonstrated pathways according to the literature.

By integrating the findings from the literature and this study, the role of PCP could be included into our previously postulated pathway of DSP toxin production (Figure 5). This light-harvesting complex might aid DSP toxin biosynthesis via various ways such as activation of some intracellular processes related to toxin biosynthesis and transportation, and also enhancements of the yields of raw materials for toxin production through absorbing extra light energy. Although the high expression of PCP isomers in the toxic strain of *P. hoffmannianum* provided us a hint that light harvesting seems to play a significant role in DSP toxin production, further investigation is required to unveil the underlying mechanisms. More in-depth physiological and molecular studies on DSP toxin-producing *Prorocentrum* species under various intensities of light and light-dark cycle could be conducted in the future.

## 3. Materials and Methods

### 3.1. Sources and Maintenance of Dinoflagellates

The toxic strain CCMP2804 and non-toxic strain CCMP683 of *P. hoffmannianum* were obtained from the National Center for Marine Algae and Microbiota, Maine, USA. They were originally isolated in Marquesas Keys and Knights Key respectively within Florida, USA. Both strains of *P. hoffmannianum* were grown in autoclaved L1 medium without sodium silicate (L1-Si medium) in Erlenmeyer flasks [61]. The medium was prepared using filtered seawater with salinity adjusted to 30 ppt. The nitrate and phosphate concentrations in the medium were 883 μM and 36.2 μM, respectively. All cultures were maintained in a growth chamber at a constant temperature of 22 °C under a 12:12 h light-dark cycle. Illumination was provided by cool white fluorescent lamps with light intensity of 3000 lx. Stock cultures were maintained in log phase of growth by transferring previous cultures into new L1-Si medium in a ratio of 1:10 *v*/*v* every three weeks.

### 3.2. Cultivation Conditions and Cell Harvesting

Each 2 mL aliquot from the stock cultures of both strains during late log phase was stained with 20 μL Lugol’s solution and the mixed sample was filled to a Sedgewick-Rafter counting chamber. After all algal cells settled and were only observed at the bottom of the chamber, they were counted directly under a light microscope from 100 squares. Only cells touching the top and left borders of each square were counted if they fall on the grid lines. For comparative proteomics under normal culture condition, 10^5^ cells of each strain were inoculated into L1-Si medium in triplicate such that a cell density of 10^3^ cells·mL^−1^ in a total volume of 100 mL was achieved. The inoculated cells were allowed to grow in a growth chamber under the same environment described in Section 3.1 and were harvested on day 28 when both strains showed similar growth rates in log phase as revealed in our previous study [33]. Apart from normal L1-Si medium, cells were inoculated into L1-Si medium depleted of phosphate with only negligible level of phosphate in natural seawater. All other growth parameters were followed as previously mentioned except that the cultivation lasted longer until day 50 when the largest discrepancy in toxicity between CCMP2804 and CCMP683 was observed [33]. Cells were harvested by centrifugation at 1620× *g* for 3 min at room temperature. After discarding the medium, the cell pellet was washed twice with autoclaved seawater and then centrifuged at 9400× *g* for 1 min. The supernatant was completely removed and the pellet was stored at −80 °C before protein extraction.

### 3.3. Proteome Analysis

#### 3.3.1. Protein Extraction and Quantification

TRIzol method with a commercial clean up kit was found to be desirable for protein extraction from *P. hoffmannianum* as shown in a previous study where high-quality 2-DE images were obtained with no significant drop in protein yield after clean up [62]. The pellet was firstly extracted by TRIzol method according to the procedures mentioned until the protein precipitate was completely dissolved in lysis buffer. The procedure of protein extraction using TRIzol reagent (Life Technologies, Carlsbad, CA, USA) was according to manufacturer’s instruction with some modification mentioned in Lee’s study [63]. The dissolved protein was further purified using a commercial 2-D clean up kit (GE Healthcare, Chicago, IL, USA) according to the manufacturer’s manual.

The amount of extracted protein was determined by a modified Bradford assay using a commercial Bradford reagent (Bio-Rad, Hercules, CA, USA) [63]. This assay was performed in 96-well microplate and in triplicate. First, 10 μL of each protein sample in lysis buffer was diluted in 190 μL deionized water. Then, 50 μL Bradford reagent was added to each well and mixed well with a multichannel pipette. After incubation at room temperature for 15 min, the absorbance was measured at 595 nm using Multiskan™ GO Microplate Spectrophotometer. Lysis buffer acted as a blank to eliminate the absorbance of urea.

#### 3.3.2. Two-Dimensional Gel Electrophoresis and Image Analysis

The range of immobilized pH gradient (IPG) and percentage of SDS-PAGE used for 2-DE were pH 4–7 and 10% respectively. Such pH range was consistent with the range of pI of proteins in many dinoflagellates reported in the literature [24,25,34,64,65]. In first dimension of 2-DE, each IPG strip was rehydrated in 340 μL rehydration buffer (containing 7 M urea, 2 M thiourea, 4% CHAPS, 0.2% dithiothreitol (DTT) and 3.4 μL IPG buffer pH 3–10) for 16 h. 100 μg protein from each sample was mixed with rehydration buffer before rehydration. IEF was performed using a Protean IEF cell (Bio-Rad, Hercules, CA, USA). The program of voltage applied was according to the following: 100 V 1 h, 300 V 2 h, 1000 V 2 h, 4000 V 2 h and 8000 V 5 h. After IEF, the gel strip was equilibrated with equilibration buffer (50 mM Tris pH 8.8, 6 M urea, 30% glycerol, 2% SDS, and 1% DTT) for 30 min. The IEF gel was then incubated with equilibration buffer containing 1% iodoacetamine (IAA) for 30 min. In the second dimension, proteins were then separated by 10% SDS-PAGE at 15 mA. Protein spots in the 2-DE gel were visualized by silver staining. Protein spots were then analyzed by using Melanie 7 (GeneBio, Geneva, Switzerland) according to the user manual.

#### 3.3.3. In-Gel Digestion

Differentially expressed protein spots (with at least two-fold differences in term of intensity) were selected. Protein spots of interest were excised from the 2-DE gels and destained by destain solution (with 0.01 g·mL^−1^ of potassium ferricyanide and 0.016 g·mL^−1^ of sodium thiosulfate). The excised gels were washed twice with 25 mM ammonium bicarbonate for 5 min in each wash. The gels were further washed with 25 mM ammonium bicarbonate in 50% acetonitrile for 5 min. The gels would then become colorless and were dehydrated by adding 100% acetonitrile. The dehydrated gels were then subjected to reduction (incubated with 10mM DTT at 55 °C for 45 min) and alkylation (incubation with 10 mM IAA at room temperature in dark for 45 min). The gel pieces were then washed again with 25 mM ammonium bicarbonate in 50% acetonitrile and dried by 100% acetonitrile again. For in-gel trypsin digestion, the dried gel pieces were incubated with freshly prepared trypsin (Promega, Road Madison, WI, USA) solution (20 ng·mL^−1^) at 37 °C overnight. Digested peptides were eluted from the gel pieces by adding the elution buffer (0.1% triflouroacetic acid in 50% acetonitrile) with the aid of ultrasonication. The eluted peptides would be stored at −80 °C until use.

#### 3.3.4. MALDI-TOF Mass Spectrometry (MS) Analysis

α-cyano-4-hydroxycinnamic acid (HCCA) was used as the matrix. 2 mg·mL^−1^ of saturated HCCA in 0.1% triflouroacetic acid with acetonitrile was coated on each spot on the target plate—anchor-chip (Bruker, Karlsruhe, Germany). For each sample, 1 μL of the dried peptides (resuspended in 2 μL of 0.1% triflouroacetic acid with acetonitrile) was added onto the dried anchor spots. Solution of the anchor spots was air dried. The dried samples were recrystallized with 1 μL recrystallization solution (Ethanol: Acetone: 0.1% TFA = 6:3:1). The samples were then analyzed by using reflector mode of Bruker Autoflex III Series High-Performance MALDI-TOF and TOF/TOF Systems. Calibrations were performed by external peptide calibration standard (Bruker, Karlsruhe, Germany ). Peptide-mass-fingerprint (PMF) profile for each sample was generated by combining 1000 shots at several different positions on the corresponding spot. All PMT profiles were searched against the NCBI database using the search engine MASCOT [66].

#### 3.3.5. De Novo Peptide Sequencing and N-Terminal Sulfonation

The amino acid sequences were derived from generated MS/MS spectra by using the de novo sequencing function of the software BioTools^TM^ 3.2 (Bruker, Karlsruhe, Germany). The identity of unknown protein could be found by searching the NCBI database using the search engine MASCOT. N-terminal sulfonation prior to MS/MS analysis could facilitate the de novo peptides sequencing. In this approach, some additional steps were introduced after in-gel digestion. Sulfonation were performed by incubating the tryptic peptide with 10 mg·mL^−1^ 4-sulfophenyl isothiocyanate (SPITC) in 20mM sodium bicarbonate at pH 9.5 in 55 °C for 30 min. This reaction was stopped with 0.5% TFA. The sulphonated peptides were cleaned by absorption onto and subsequently eluted from C18 zip-tips. The eluted samples were directly introduced onto the HCCA coated anchor spots. De novo peptide sequencing was performed as previously described while an additional 215 Da of mass would appear in successfully sulfonated peptides.

## Figures and Tables

**Table 1:** Summary of identified proteins after de novo peptide sequencing.

Spots	Fragment Peaks (*m*/*z*)	Protein Identity	Differential Expression
T2b	1229.6	Putative uncharacterized protein	Only expressed in CCMP2804 compared to CCMP683 under normal condition
T5b,c T6a–d T7a–d T10a–d T11a–d T12a–c TP5 TP6a,b TP7a–c TP8a–d	1258.6 2393.2	Peridinin-chlorophyll a-binding protein	Two- to four-fold upregulation or only expressed in CCMP2804 compared to CCMP683 under normal condition or phosphate depletion

## Supplementary Materials

The following supporting information can be downloaded at: https://www.mdpi.com/article/10.3390/ijms24021735/s1.
