# Supplementary material for: Upregulation of Peridinin-Chlorophyll A-Binding Protein in a Toxic Strain of Prorocentrum hoffmannianum under Normal and Phosphate-Depleted Conditions
Source: Int J Mol Sci. 2023 Jan 15;24(2):1735. doi: 10.3390/ijms24021735 (PMC9864435; doi:10.3390/ijms24021735)
Supplement: Supplementary file 1 [file ijms-24-01735-s001.zip › ijms-2144201-supplementary.pdf]

Supplementary Materials

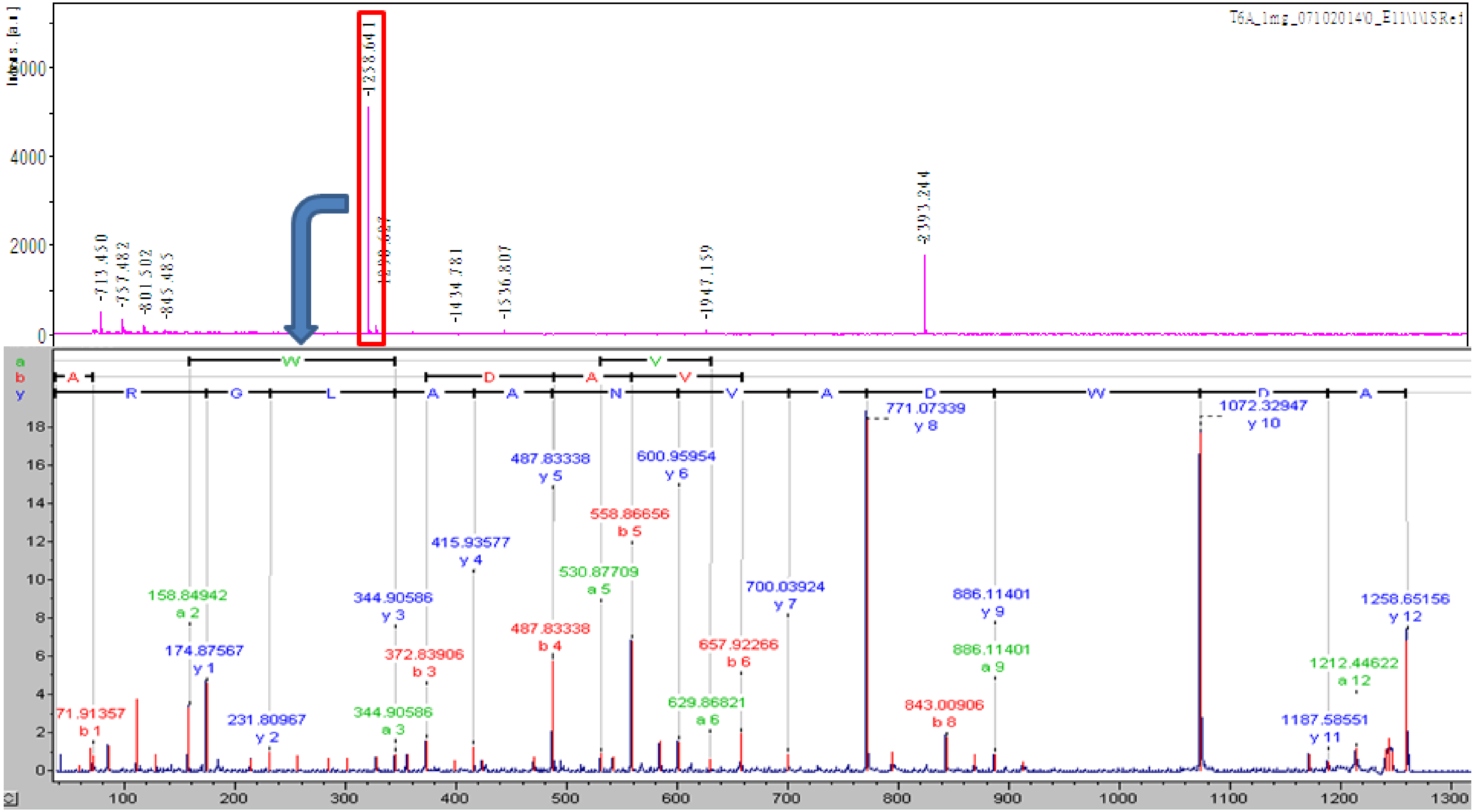

Figure S1. MS/MS spectrum of peptide m/z 1258.6 from spot T6a. The sequence ADWDAVNAALGR was deduced from the y-ion with mass tolerance of ±0.3 Da.

Table S1. Information of up-regulated protein spots found in the 2-DE profiles of CCMP2804 or CCMP683 when compared to each other under normal culture condition.

Protein Spot [1]	Isoelectric Point	Molecular Mass (kDa)	Folds of Upregulation [2]
T1a	6.2	85	n/a
T1b	6.3	85	n/a
T2a	5.3	55	6.2
T2b	5.4	55	n/a
T3	6.4	53	n/a
T4	4.6	41	2.2
T5a	6.1	34	n/a
T5b	6.1	32	2
T5c	6.1	31	2
T6a	5.8	36	2
T6b	5.8	34	2
T6c	5.8	32	2
T6d	5.8	31	3
T7a	5.5	34	2
T7b	5.5	33	2.4
T7c	5.5	32	3.9
T7d	5.5	31	3.6
T8	6.25	25	n/a
T9	5.4	32	n/a
T10a	5.3	36	n/a
T10b	5.3	34	n/a
T10c	5.3	32	2
T10d	5.3	31	2
T11a	5.2	36	n/a
T11b	5.2	35	n/a
T11c	5.2	33	n/a
T11d	5.2	32	2
T12a	5.1	35	2.97
T12b	5.1	34	n/a
T12c	5.1	32	2
T13	4.8	25	n/a
T14	5.05	18	n/a
T15	4.5	19	n/a
NT1a	6.3	90	n/a
NT1b	6.4	90	n/a
NT1c	6.45	90	n/a
NT2	5.4	67	5
NT3a	4.9	58	n/a
NT3b	5.05	58	2.2
NT4	6.45	55	4
NT5	6.15	35	3

[1] The protein spots with an initial "T" were up-regulated in the toxic strain CCMP2804 while those with an initial "NT" were up-regulated in the non-toxic strain CCMP683.

[2] The folds of change for some spots were not available because they were only observed in the 2-DE profiles of one strain.

Table S2. Information of up-regulated protein spots found in the 2-DE profiles of CCMP2804 or CCMP683 when compared to each other under phosphate depletion.

Protein Spot [1]	Isoelectric Point	Molecular Mass (kDa)	Folds of Upregulation [2]
TP1a	6.2	85	2
TP1b	6.3	85	n/a
TP2a	5.3	55	4
TP2b	5.4	55	5
TP3	6.4	53	n/a
TP4	5.5	41	n/a
TP5	6.1	34	2
TP6a	5.8	34	n/a
TP6b	5.8	33	2
TP7a	5.5	32	n/a
TP7b	5.5	30	n/a
TP7c	5.5	29	4
TP8a	5.1	34	2
TP8b	5.1	32	2
TP8c	5.1	30	3
TP8d	5.2	33	2
TP9	4.8	25	n/a
TP10	4.3	25	n/a
TP11	4.4	23	n/a
TP12	5.4	21	n/a
NTP1a	6.3	90	4
NTP1b	6.4	90	4
NTP1c	6.45	90	n/a
NTP2a	4.9	58	n/a
NTP2b	5.05	58	n/a
NTP3	6.45	55	n/a
NTP4	6	54	n/a
NTP5	5.9	43	n/a
NTP6	5.7	42	n/a
NTP7	6.15	35	4
NTP8	6.1	30	n/a
NTP9a	4.9	26	n/a
NTP9b	4.9	26	n/a

[1] The protein spots with an initial "TP" were up-regulated in the toxic strain CCMP2804 while those with an initial "NTP" were up-regulated in the non-toxic strain CCMP683.
[2] The folds of change for some spots were not available because they were only observed in the 2-DE profiles of one strain.
